# 3-(3,4-Dichloro­benzyl­idene)chroman-4-one

**DOI:** 10.1107/S1600536812040561

**Published:** 2012-10-03

**Authors:** Kaalin Gopaul, Neil Anthony Koorbanally, Mahidansha M. Shaikh, Hong Su, Deresh Ramjugernath

**Affiliations:** a School of Chemistry and Physics, University of KwaZulu-Natal, Private Bag X54001, Durban 4000, South Africa; b Chemistry Department, University of Cape Town, Rondebosch, 7701, South Africa; c School of Chemical Engineering, University of KwaZulu-Natal, Durban 4041, South Africa

## Abstract

The distinctive feature of the structure of the title compound, C_16_H_10_Cl_2_O_2_, is the formation of a zigzag chain along [100] *via* Cl⋯Cl inter­actions [3.591 (1) and 3.631 (1) Å]. The chroman­one moiety is fused with the benzene ring and adopts a half-chair conformation. The dihedral angle between the benzene ring of the chromanone moiety and the dichlorobenzene plane is 56.14 (8)°.

## Related literature

For background to homoisoflavonoids, see: Kirkiacharian *et al.* (1984 ▶). For a related structure, see: Gopaul *et al.* (2012 ▶).
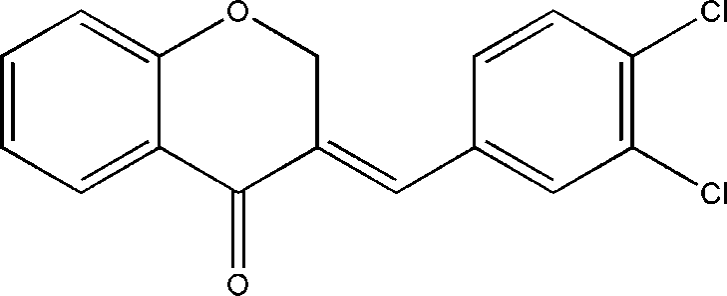


## Experimental

### 

#### Crystal data

C_16_H_10_Cl_2_O_2_
*M* *_r_* = 305.14
Monoclinic, 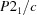

*a* = 3.9224 (3) Å
*b* = 11.5175 (10) Å
*c* = 28.957 (3) Å
β = 92.270 (2)°
*V* = 1307.12 (19) Å^3^
*Z* = 4
Mo *K*α radiation
μ = 0.49 mm^−1^
*T* = 173 K
0.16 × 0.12 × 0.11 mm

#### Data collection

Bruker Kappa Duo APEXII Diffractometer
Absorption correction: multi-scan (*SADABS*; Sheldrick, 1997 ▶) *T*_min_ = 0.925, *T*_max_ = 0.948
15291 measured reflections
3258 independent reflections
2611 reflections with *I* > 2σ(*I*)
*R*_int_ = 0.037

#### Refinement

*R*[*F* ^2^ > 2σ(*F* ^2^)] = 0.034
*wR*(*F* ^2^) = 0.089
*S* = 1.03
3258 reflections
181 parameters
H-atom parameters constrained
Δρ_max_ = 0.30 e Å^−3^
Δρ_min_ = −0.21 e Å^−3^

### 

Data collection: *APEX2* (Bruker, 2006 ▶); cell refinement: *SAINT* (Bruker, 2006 ▶); data reduction: *SAINT*; program(s) used to solve structure: *SHELXS97* (Sheldrick, 2008 ▶); program(s) used to refine structure: *SHELXL97* (Sheldrick, 2008 ▶); molecular graphics: *ORTEP-3* (Farrugia, 2012 ▶); software used to prepare material for publication: *SHELXL97*.

## Supplementary Material

Crystal structure: contains datablock(s) I, global. DOI: 10.1107/S1600536812040561/hg5252sup1.cif

Structure factors: contains datablock(s) I. DOI: 10.1107/S1600536812040561/hg5252Isup2.hkl

Additional supplementary materials:  crystallographic information; 3D view; checkCIF report

## Figures and Tables

**Fig. 1.**
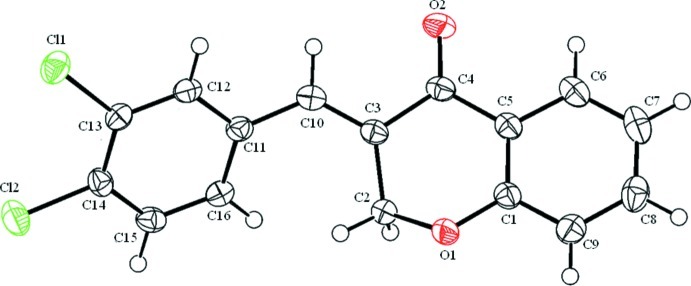
Molecular structure of the title compound, showing the atom labelling scheme and with displacement ellipsoids drawn at the 50% probability level.

**Fig. 2.**
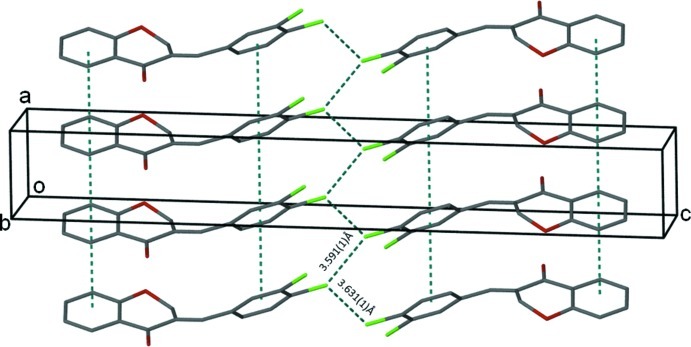
Partial packing diagram showing the Cl···Cl interactions and π···π stacking as dashed lines. H atoms have been omitted.

## supplementary crystallographic information

### Comment

The title compound, 3-(3,4-dichlorobenzylidene)chroman-4-one (C_16_H_10_Cl_2_O_2_), belongs to a class of compounds called homoisoflavonoids, which are C-16, *α*,*β* unsaturated carbonyl compounds containing two aromatic rings. They are a group of naturally occurring molecules that are structurally related to isoflavonoids but differ by containing one more carbon atom (Kirkiacharian *et al.*, 1984; Gopaul *et al.*, 2012).

A view of the the title compound is shown in Fig. 1. The chromanone moiety is fused with the benzene ring and adopts a half chair conformation. The dihedral angle between the benzene ring of the chromanone moiety and the dichlorobenzene plane is 56.14 (8)°. The inversion-related molecules are linked into zigzag chains *via* Cl···Cl interactions between Cl2 at (*x*, *y*, *z*) and Cl2 at (1 - *x*, 1 - *y*, 1 - *z*) and Cl2 at (-*x*, 1 - *y*, 1 - *z*) with distances of 3.591 (1) Å and 3.631 (1) Å respectively. Molecules related by translation along the *a* axis stack *via* double π···π interactions of the aromatic rings, with a centroid distance equal to the length of *a* axis. This feature is illustrated in Fig. 2.

### Experimental

A mixture of chroman-4-one (1 g, 6.749 mmol), 3,4-dichlorobenzaldehyde (1.417 g, 8.099 mmol) and 10–15 drops of piperidine was heated at 80°C for 18 hrs. The reaction mixture was monitored for completion by thin layer chromatography. Upon completion, the reaction mixture was cooled, diluted with water and neutralized using 10% HCl. The reaction mixture was extracted with ethyl acetate (3 × 30 ml). The ethyl acetate layers were combined, washed with brine (20 ml), water (2 × 10 ml) and dried over anhydrous magnesium sulfate. The solvent was reduced and the compound purified by column chromatography using silica gel (Merck 9385, 40–63 *µ*m particle size) with a mobile phase of 2% ethyl acetate in hexane to yield the title compound with a m.p. of 165–167 °C.

^1^H NMR: *δ* (p.p.m.): 5.27 (2*H*, d, *J* = 1.88 Hz, H-2), 6.96 (1*H*, d, *J* = 8.28 Hz, H-8), 7.07 (1*H*, td, *J* = 7.52, 0.68 Hz, H-6), 7.12 (1*H*, dd, *J* = 8.28, 1.96 Hz, H-6'), 7.38 (1*H*, d, *J* = 1.92 Hz, H-2'), 7.49 (1*H*, ddd, *J* = 8.72, 7.48, 1.72 Hz, H-7), 7.50 (1*H*, d, *J* = 8.40 Hz, H-5'), 7.72 (1*H*, s, H-9), 8.00 (1*H*, dd, *J* = 7.88, 1.64 Hz, H-5)

^13^C NMR: *δ* (p.p.m.): 67.27 (C-2), 118.00 (C-8), 121.79 (C-4a), 122.16 (C-6), 128.00 (C-5), 128.94 (C-6'), 130.79 (C-5'), 131.41 (C-2'), 132.44 (C-3), 133.15 (C-1'), 133.69 (C-3'), 134.28 (C-4'), 134.55 (C-9), 136.19 (C-7), 161.13 (C-8a), 181.71(C-4)

### Refinement

All hydrogen atoms were placed in geometrically idealized positions and constrainted to ride on their parent atoms, with aromatic C—H = 0.95 Å and methylene C—H = 0.99 Å; *U*_iso_(H) = 1.2*U*_eq_(C).

### Crystal data

**Table:**

C_16_H_10_Cl_2_O_2_	*F*(000) = 624
*M**_r_* = 305.14	*D*_x_ = 1.551 Mg m^−^^3^
Monoclinic, *P*2_1_/*c*	Mo *K*α radiation, λ = 0.71073 Å
Hall symbol: -P 2ybc	Cell parameters from 15291 reflections
*a* = 3.9224 (3) Å	θ = 1.9–28.3°
*b* = 11.5175 (10) Å	µ = 0.49 mm^−^^1^
*c* = 28.957 (3) Å	*T* = 173 K
β = 92.270 (2)°	Block, colourless
*V* = 1307.12 (19) Å^3^	0.16 × 0.12 × 0.11 mm
*Z* = 4	

### Data collection

**Table:**

Bruker Kappa Duo APEXII Diffractometer	3258 independent reflections
Radiation source: fine-focus sealed tube	2611 reflections with *I* > 2σ(*I*)
Graphite monochromator	*R*_int_ = 0.037
0.5° φ scans and ω scans	θ_max_ = 28.3°, θ_min_ = 1.9°
Absorption correction: multi-scan (*SADABS*; Sheldrick, 1997)	*h* = −5→5
*T*_min_ = 0.925, *T*_max_ = 0.948	*k* = −15→15
15291 measured reflections	*l* = −38→38

### Refinement

**Table:**

Refinement on *F*^2^	Primary atom site location: structure-invariant direct methods
Least-squares matrix: full	Secondary atom site location: difference Fourier map
*R*[*F*^2^ > 2σ(*F*^2^)] = 0.034	Hydrogen site location: inferred from neighbouring sites
*wR*(*F*^2^) = 0.089	H-atom parameters constrained
*S* = 1.03	*w* = 1/[σ^2^(*F*_o_^2^) + (0.0416*P*)^2^ + 0.370*P*] where *P* = (*F*_o_^2^ + 2*F*_c_^2^)/3
3258 reflections	(Δ/σ)_max_ = 0.001
181 parameters	Δρ_max_ = 0.30 e Å^−^^3^
0 restraints	Δρ_min_ = −0.21 e Å^−^^3^

### Special details

**Table:**

Geometry. All e.s.d.'s (except the e.s.d. in the dihedral angle between two l.s. planes) are estimated using the full covariance matrix. The cell e.s.d.'s are taken into account individually in the estimation of e.s.d.'s in distances, angles and torsion angles; correlations between e.s.d.'s in cell parameters are only used when they are defined by crystal symmetry. An approximate (isotropic) treatment of cell e.s.d.'s is used for estimating e.s.d.'s involving l.s. planes.
Refinement. Refinement of *F*^2^ against ALL reflections. The weighted *R*-factor *wR* and goodness of fit *S* are based on *F*^2^, conventional *R*-factors *R* are based on *F*, with *F* set to zero for negative *F*^2^. The threshold expression of *F*^2^ > σ(*F*^2^) is used only for calculating *R*-factors(gt) *etc*. and is not relevant to the choice of reflections for refinement. *R*-factors based on *F*^2^ are statistically about twice as large as those based on *F*, and *R*- factors based on ALL data will be even larger.

### Fractional atomic coordinates and isotropic or equivalent isotropic displacement parameters (Å^2^)

**Table:**

	*x*	*y*	*z*	*U*_iso_*/*U*_eq_	
Cl1	0.42734 (11)	0.87066 (4)	0.446666 (14)	0.03559 (12)	
Cl2	0.24634 (12)	0.61056 (4)	0.471571 (14)	0.04001 (13)	
O1	0.1216 (3)	0.60873 (9)	0.19421 (4)	0.0295 (3)	
O2	−0.4016 (3)	0.91508 (10)	0.20528 (4)	0.0370 (3)	
C1	0.0018 (4)	0.66516 (13)	0.15546 (5)	0.0257 (3)	
C2	−0.0674 (4)	0.62604 (13)	0.23556 (5)	0.0264 (3)	
H2A	0.0689	0.5962	0.2625	0.032*	
H2B	−0.2815	0.5806	0.2330	0.032*	
C3	−0.1530 (4)	0.75127 (12)	0.24381 (5)	0.0246 (3)	
C4	−0.2625 (4)	0.82037 (13)	0.20229 (6)	0.0263 (3)	
C5	−0.1875 (4)	0.76778 (13)	0.15740 (5)	0.0253 (3)	
C6	−0.2890 (4)	0.82258 (14)	0.11584 (6)	0.0316 (4)	
H6	−0.4186	0.8922	0.1166	0.038*	
C7	−0.2037 (5)	0.77697 (16)	0.07401 (6)	0.0374 (4)	
H7	−0.2737	0.8148	0.0461	0.045*	
C8	−0.0143 (5)	0.67520 (16)	0.07285 (6)	0.0365 (4)	
H8	0.0453	0.6437	0.0439	0.044*	
C9	0.0882 (4)	0.61927 (14)	0.11313 (6)	0.0315 (3)	
H9	0.2174	0.5496	0.1120	0.038*	
C10	−0.1366 (4)	0.80455 (13)	0.28501 (5)	0.0269 (3)	
H10	−0.1923	0.8848	0.2848	0.032*	
C11	−0.0434 (4)	0.75431 (13)	0.33026 (5)	0.0253 (3)	
C12	0.1296 (4)	0.82387 (13)	0.36316 (5)	0.0258 (3)	
H12	0.1865	0.9015	0.3555	0.031*	
C13	0.2187 (4)	0.78142 (13)	0.40650 (5)	0.0256 (3)	
C14	0.1346 (4)	0.66795 (14)	0.41804 (5)	0.0263 (3)	
C15	−0.0449 (4)	0.59937 (13)	0.38621 (6)	0.0275 (3)	
H15	−0.1075	0.5226	0.3944	0.033*	
C16	−0.1336 (4)	0.64150 (13)	0.34272 (5)	0.0261 (3)	
H16	−0.2565	0.5936	0.3212	0.031*	

### Atomic displacement parameters (Å^2^)

**Table:**

	*U*^11^	*U*^22^	*U*^33^	*U*^12^	*U*^13^	*U*^23^
Cl1	0.0386 (2)	0.0369 (2)	0.0310 (2)	−0.00723 (17)	−0.00110 (17)	−0.00681 (16)
Cl2	0.0470 (3)	0.0436 (3)	0.0291 (2)	−0.00285 (19)	−0.00218 (18)	0.00968 (17)
O1	0.0354 (6)	0.0268 (6)	0.0265 (6)	0.0085 (4)	0.0024 (5)	0.0026 (4)
O2	0.0447 (7)	0.0258 (6)	0.0400 (7)	0.0113 (5)	−0.0050 (6)	0.0018 (5)
C1	0.0265 (7)	0.0234 (7)	0.0269 (8)	−0.0051 (6)	−0.0014 (6)	0.0021 (6)
C2	0.0314 (8)	0.0232 (7)	0.0245 (7)	0.0025 (6)	0.0013 (6)	0.0011 (6)
C3	0.0226 (7)	0.0227 (7)	0.0283 (8)	0.0016 (5)	−0.0007 (6)	0.0020 (6)
C4	0.0247 (7)	0.0217 (7)	0.0321 (8)	0.0006 (6)	−0.0032 (6)	0.0020 (6)
C5	0.0236 (7)	0.0240 (7)	0.0279 (8)	−0.0047 (6)	−0.0030 (6)	0.0034 (6)
C6	0.0283 (8)	0.0314 (8)	0.0348 (9)	−0.0041 (6)	−0.0046 (7)	0.0082 (7)
C7	0.0363 (9)	0.0467 (10)	0.0286 (9)	−0.0110 (8)	−0.0049 (7)	0.0108 (7)
C8	0.0387 (9)	0.0440 (10)	0.0271 (8)	−0.0118 (8)	0.0044 (7)	−0.0016 (7)
C9	0.0332 (8)	0.0298 (8)	0.0317 (9)	−0.0066 (7)	0.0052 (7)	−0.0013 (6)
C10	0.0262 (7)	0.0224 (7)	0.0322 (8)	0.0013 (6)	0.0014 (6)	0.0013 (6)
C11	0.0264 (7)	0.0232 (7)	0.0265 (8)	0.0030 (6)	0.0044 (6)	−0.0008 (6)
C12	0.0282 (8)	0.0207 (7)	0.0287 (8)	0.0000 (6)	0.0052 (6)	−0.0021 (6)
C13	0.0242 (7)	0.0266 (7)	0.0261 (8)	−0.0008 (6)	0.0030 (6)	−0.0038 (6)
C14	0.0258 (7)	0.0299 (8)	0.0233 (7)	0.0020 (6)	0.0036 (6)	0.0039 (6)
C15	0.0274 (8)	0.0224 (7)	0.0330 (8)	−0.0016 (6)	0.0058 (6)	0.0021 (6)
C16	0.0257 (7)	0.0239 (7)	0.0287 (8)	−0.0010 (6)	0.0020 (6)	−0.0032 (6)

### Geometric parameters (Å, º)

**Table:**

Cl1—C13	1.7330 (15)	C7—C8	1.389 (3)
Cl2—C14	1.7256 (15)	C7—H7	0.9500
O1—C1	1.3641 (18)	C8—C9	1.378 (2)
O1—C2	1.4470 (19)	C8—H8	0.9500
O2—C4	1.2243 (19)	C9—H9	0.9500
C1—C9	1.389 (2)	C10—C11	1.465 (2)
C1—C5	1.398 (2)	C10—H10	0.9500
C2—C3	1.502 (2)	C11—C16	1.398 (2)
C2—H2A	0.9900	C11—C12	1.400 (2)
C2—H2B	0.9900	C12—C13	1.379 (2)
C3—C10	1.341 (2)	C12—H12	0.9500
C3—C4	1.491 (2)	C13—C14	1.392 (2)
C4—C5	1.474 (2)	C14—C15	1.385 (2)
C5—C6	1.402 (2)	C15—C16	1.381 (2)
C6—C7	1.374 (3)	C15—H15	0.9500
C6—H6	0.9500	C16—H16	0.9500
			
C1—O1—C2	116.35 (12)	C9—C8—H8	119.6
O1—C1—C9	117.15 (14)	C7—C8—H8	119.6
O1—C1—C5	122.42 (14)	C8—C9—C1	119.71 (16)
C9—C1—C5	120.37 (15)	C8—C9—H9	120.1
O1—C2—C3	112.89 (12)	C1—C9—H9	120.1
O1—C2—H2A	109.0	C3—C10—C11	128.05 (14)
C3—C2—H2A	109.0	C3—C10—H10	116.0
O1—C2—H2B	109.0	C11—C10—H10	116.0
C3—C2—H2B	109.0	C16—C11—C12	118.51 (14)
H2A—C2—H2B	107.8	C16—C11—C10	122.76 (14)
C10—C3—C4	118.39 (13)	C12—C11—C10	118.66 (13)
C10—C3—C2	125.29 (14)	C13—C12—C11	121.01 (14)
C4—C3—C2	116.32 (13)	C13—C12—H12	119.5
O2—C4—C5	122.25 (14)	C11—C12—H12	119.5
O2—C4—C3	122.24 (15)	C12—C13—C14	119.84 (14)
C5—C4—C3	115.51 (13)	C12—C13—Cl1	119.77 (12)
C1—C5—C6	118.63 (15)	C14—C13—Cl1	120.38 (12)
C1—C5—C4	120.50 (13)	C15—C14—C13	119.66 (14)
C6—C5—C4	120.79 (14)	C15—C14—Cl2	118.93 (12)
C7—C6—C5	120.90 (16)	C13—C14—Cl2	121.41 (12)
C7—C6—H6	119.5	C16—C15—C14	120.64 (14)
C5—C6—H6	119.5	C16—C15—H15	119.7
C6—C7—C8	119.56 (16)	C14—C15—H15	119.7
C6—C7—H7	120.2	C15—C16—C11	120.31 (14)
C8—C7—H7	120.2	C15—C16—H16	119.8
C9—C8—C7	120.82 (16)	C11—C16—H16	119.8
			
C2—O1—C1—C9	−156.47 (14)	C7—C8—C9—C1	−0.1 (2)
C2—O1—C1—C5	26.3 (2)	O1—C1—C9—C8	−177.45 (14)
C1—O1—C2—C3	−46.21 (17)	C5—C1—C9—C8	−0.2 (2)
O1—C2—C3—C10	−138.89 (16)	C4—C3—C10—C11	178.67 (15)
O1—C2—C3—C4	40.74 (18)	C2—C3—C10—C11	−1.7 (3)
C10—C3—C4—O2	−15.1 (2)	C3—C10—C11—C16	−36.4 (2)
C2—C3—C4—O2	165.28 (15)	C3—C10—C11—C12	146.59 (17)
C10—C3—C4—C5	164.10 (14)	C16—C11—C12—C13	1.9 (2)
C2—C3—C4—C5	−15.56 (19)	C10—C11—C12—C13	179.04 (14)
O1—C1—C5—C6	177.47 (13)	C11—C12—C13—C14	−0.4 (2)
C9—C1—C5—C6	0.3 (2)	C11—C12—C13—Cl1	−179.05 (12)
O1—C1—C5—C4	0.7 (2)	C12—C13—C14—C15	−1.4 (2)
C9—C1—C5—C4	−176.41 (14)	Cl1—C13—C14—C15	177.26 (12)
O2—C4—C5—C1	173.56 (15)	C12—C13—C14—Cl2	179.08 (12)
C3—C4—C5—C1	−5.6 (2)	Cl1—C13—C14—Cl2	−2.23 (19)
O2—C4—C5—C6	−3.1 (2)	C13—C14—C15—C16	1.6 (2)
C3—C4—C5—C6	177.72 (14)	Cl2—C14—C15—C16	−178.85 (12)
C1—C5—C6—C7	−0.3 (2)	C14—C15—C16—C11	−0.1 (2)
C4—C5—C6—C7	176.48 (15)	C12—C11—C16—C15	−1.7 (2)
C5—C6—C7—C8	0.0 (2)	C10—C11—C16—C15	−178.69 (15)
C6—C7—C8—C9	0.2 (3)		
